# Assessment of the Short Grit Scale in patients with substance use disorder: Reliability and patient characteristics

**DOI:** 10.1192/j.eurpsy.2023.828

**Published:** 2023-07-19

**Authors:** M. Griffin, R. Weiss, C. Trinh

**Affiliations:** 1 Harvard Medical School, Belmont; 2 University of Rhode Island, Providence, United States

## Abstract

**Introduction:**

Recovery from substance use disorder requires sustained effort and perseverance. Grit is a resilience factor that may be important for people in recovery. Little research has been conducted on grit in patients with substance use disorder, especially in a large and varied sample.

**Objectives:**

To examine the Short Grit Scale (Grit-S) in patients with substance use disorder, our aims were to analyze its psychometric properties and use demographic and clinical characteristics to predict variance in Grit-S scores.

**Methods:**

In this study of patients in treatment for substance use disorder, participants completed the Grit-S and other self-report measures. The psychometric properties of the Grit-S were assessed in outpatients (N=94) and a hierarchical regression was used to predict Grit-S variance in inpatients (N=1238).

**Results:**

The Grit-S demonstrated good internal consistency (α=.75) and strong test-retest reliability (unadjusted r=.81, adjusted r=.79, p values<.001). The mean Grit-S score was 3.15, which was lower than other clinical samples reported in the literature. Regression modeling indicated a moderate, statistically significant association between demographic and clinical characteristics and Grit-S scores (R^2^=15.5%, p<.001).

**Conclusions:**

Of particular interest, the positive factor of recovery protection showed the strongest association with grit of all the variables assessed. Hence the positive construct of grit was correlated with other positive constructs, as well as with risks. Longitudinal assessment of grit and substance use could measure the stability and clinical significance of grit throughout recovery.

**Disclosure of Interest:**

M. Griffin: None Declared, R. Weiss Consultant of: Analgesic Solutions, WCG, & Alkermes, C. Trinh: None Declared

